# Techno-Economic Comparison of Integration Options for an Oxygen Transport Membrane Unit into a Coal Oxy-Fired Circulating Fluidized Bed Power Plant

**DOI:** 10.3390/membranes12121224

**Published:** 2022-12-02

**Authors:** E. Portillo, Luz M. Gallego Fernández, M. Cano, B. Alonso-Fariñas, B. Navarrete

**Affiliations:** Departamento de Ingeniería Química y Ambiental, Escuela Técnica Superior de Ingeniería, Universidad de Sevilla, C/Camino de los Descubrimientos s/n, 41092 Sevilla, Spain

**Keywords:** oxy-fuel combustion, circulating fluidized bed boiler, oxygen transport membrane, techno-economic

## Abstract

The inclusion of membrane-based oxygen-fired combustion in power plants is considered an emerging technology that could reduce carbon emissions in a more efficient way than cryogenic oxygen-fired processes. In this paper, a techno-economic assessment was developed for a 863 MW_el,net_ power plant to demonstrate whether this CCS technique results in a reduction in efficiency losses and economic demand. Four configurations based on oxygen transport membranes were considered, while the benchmark cases were the air combustion process without CO_2_ capture and a cryogenic oxygen-fired process. The type of driving force through the membrane (3-end or 4-end), the point of integration into the oxy-fuel combustion process, the heating system, and the pollutant control system were aspects considered in this work. In comparison, the efficiency losses for membrane-based alternatives were lower than those in the cryogenic oxygen-fired process, reaching savings of up to 14% net efficiency. Regarding the specific energy consumption for CO_2_ capture, the configuration based on the oxygen transport membrane unit with 4-end mode and hot filtration presented 1.01 kW_el,net,_·h/kg_CO2 captured_ with 100% CO_2_ recovery, which is an improvement of 11% compared with the cases using cryogenic oxygen. Comparing economic aspects, the specific investment costs for cases based on the oxygen transport membrane unit varied between 2520 and 2942 $/kW_el,net_·h. This was between 39.6 and 48.2% above the investment for the reference case without carbon capture. However, its hypothetical implantation could suppose a savings of 10.7% in terms of investment cost compared with cryogenic oxygen-based case. In terms of the levelized cost of electricity and the cost of CO_2_ avoidance, the oxygen transport membrane configurations achieved more favorable results compared with the cryogenic route, reaching savings up to 14 and 38%, respectively. Although oxygen transport membrane units are currently not mature for commercial-scale applications, the results indicated that its application within carbon capture and storage technologies can be strongly competitive.

## 1. Introduction

One of the current socioeconomic and environmental challenges of our modern society is to achieve sustainable development, which can reduce the climate change experienced over the last decades. This environmental situation is aggravated by the constant emissions of greenhouse gases (GHGs) which come from industrial sectors, such as power plants, cement factories, oil refineries, blast furnaces, mining, transportation, and so on. In light of this environmental concern, the international community has responded with increasing support since the signing of the Kyoto Protocol and the recent agreements of the Climate Summit in Madrid (COP-25) [1]. The main goal of every meeting is the commitment to carry out significant reductions in greenhouse gas emissions, mainly CO_2_ emissions, until reaching levels that prevent an increase in the Earth’s temperature by more than 2 °C. To achieve this goal of reducing CO_2_ emissions, several technical measures are proposed which could counteract the climate problem in the short to medium-term: the improvement in the energy efficiency of processes, the development of renewable energy, the use of nuclear energy, and the capture, sequestration, and utilization of CO_2_.

In this work, the oxy-fired circulating fluidized bed is studied as a carbon capture technology to alleviate climate change as related to coal-based power generation. This technology is marked by the substitution of the air by an increased oxygen content comburent for fuel combustion. Nowadays, Cryogenic Air Separation Units (Cryogenic-ASU) are the most mature technology capable of satisfying the high-purity oxygen requirement for oxy-combustion conditions on a full-scale plant. The main disadvantage of this technology is both the high energy consumption and costs associated with the separation of oxygen from the air. Another oxygen separation technique has been reported in the literature based on oxygen transport membranes (OTMs) that could reduce the techno-economic penalty with proper integration into the oxy-combustion process [2,3,4,5]. According to these researchers, it is possible to achieve savings between 0.5 and 9% from a thermodynamic point of view if this alternative is implemented [5]. In terms of economic considerations, the OTM unit has been reported to achieve a cost between 0.063 and 10.58 $/kWh and between 16.7 and 57 $/tn CO_2,avoidance_, which means savings of 10.5 to 17.3% compared with the ASU unit [5,6,7]. A thermodynamic analysis using simulation models to compare different scenarios of oxy-combustion technology equipped with OTM units for O_2_ separation was developed in Portillo et al. [2]. Although their results showed energy savings of between 0.2 and 5.1% compared with the ASU unit, they did not consider economic aspects to validate their conclusions [2]. Toan Vu et al. [8] developed a techno-economic assessment of an air-fired power plant without CO_2_ capture against a post-combustion process based on an amine absorber unit and a cryogenic oxygen-fired power plant. Although their results demonstrated that oxy-combustion process was advantageous compared with the post-combustion scenario (showing costs of 59 $/MWh and 64 $/MWh, respectively), OTM units were not judged as oxygen suppliers in this research. Maas et al. [9], Castillo et al. [10], and Stadler et al. [11] presented a thermodynamic analysis in which a conventional power generation system was compared to two configurations consisting of an oxy-combustion process and an OTM unit. However, these investigations did not weigh all possible integrations between OTMs and power plant processes, and economic aspects were not considered as a comparison criterion.

Because of the results and conclusions of these trials, the need to develop technical-economic studies to compare the conventional air-combustion system with oxy-fuel plants equipped with different oxygen separation units has arisen. This work shows a techno-economic assessment of six coal-fired power plants: (1) conventional power plant without CO_2_ capture, (2) oxy-combustion process equipped by a cryogenic unit with CO_2_ capture, and (3) four configurations composed of different integrations between the oxy-fuel process and the OTM unit with CO_2_ capture. The mass and energy balance of each case has been developed through commercial process simulation software, considering they are under similar design criteria and boundary conditions. Both technical indicators and qualitative criteria have been included in the comparison, such as environmental issues, thermodynamic, and economic aspects. From a thermodynamic point of view, the net electric power, the net efficiency of electricity production, and the specific energy consumption for CO_2_ capture (SPCCC) have been selected as comparative parameters. Total capital investment (TCI), total production cost (TPC), levelized cost of electricity (LCOE), CO_2_ capture cost, and CO_2_ avoidance cost were used as economic indicators.

The aim of this study is first to identify the advantages and disadvantages of the proposed CO_2_ capture systems, providing information to identify the best technical and economical alternative to be integrated into a full-scale plant. Secondly, this work is expected to verify whether the OTM technology achieves fewer energy and economic requirements compared with the ASU unit. Finally, the membrane area (m^2^) and the specific membrane area (m^2^/kW_el,net_) are the design parameters selected to evaluate the viability of OTM units with respect to their possible location in real processes.

This paper is structured as follows: Section 2 presents the proposed scenarios, the main assumptions and input data; Section 3 describes the research methodology; the results, along with their discussion, are shown in Section 4; lastly, the main conclusions, highlights, and future research horizons are reported in Section 5.

## 2. System Configuration Description and Assumptions

The techno-economic comparison of this work was based on five anthracite coal-based CFB power plants with different CO_2_ capture alternatives. To achieve commercial plant viability, the selection criteria for the potential best alternative should involve a minimum cost related to include its system CO_2_ capture as well as to ensure a reduction in efficiency penalty. An air-fired CFB supercritical plant without CO_2_ capture was set as the Reference Case. A gross electrical power of 863 MW_el,gross_ and gross electrical efficiency of 40% were fixed as input. Table 1 presents the main features of each case assessed, with the detailed descriptions in Section 2.1 and Section 2.2. As can be seen, this work included the assumptions reported in Portillo et al. with the aim to validate the obtained results from an economic point of view [2].

### 2.1. Reference Case: Air Coal-Fired CFC Supercritical without CO_2_ Capture

**Figure 1:**
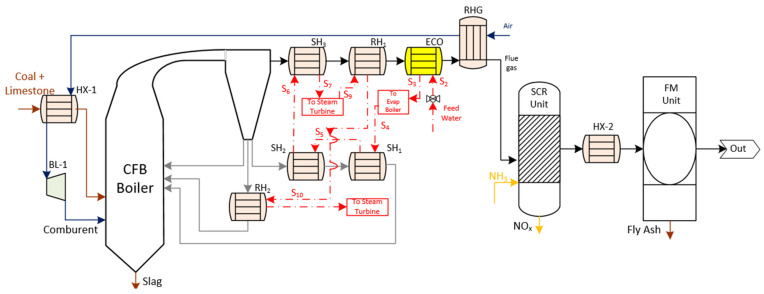
Schematic diagram of the Reference Case (Coal-fired power plant process without CO_2_ capture) adapted from [2,12,13].

The Reference Case 863 MW_el,gross_ CFB boiler (Figure 1) was characterized by the combustion of anthracite coal with air through a circulating fluidized bed that is formed of sand, limestone, coal, and combustion waste. CFB boilers allow the reduction of the temperature with a higher combustion time, thus achieving a higher stabilization of the temperature in the combustion zone and lower formation of NO_x_. Additionally, this type of boiler has great versatility in its fuels, admitting impurities, and higher particle size distribution. Thanks to the introduction of limestone in the bed, the flue gas shows lower SO_x_ concentration because desulphurization occurs in the boiler.

The boiler was divided between the hearth, the return of solids, and the convective zone. In the hearth, the coal is burned with air, which is introduced through a grate to fluidize the bed at the base. The solid material was dragged and separated from the flue gas stream in a hot separator which moved the solids downward to the loop seal through the cyclone downs-comer. The loop seal (sealing zone) had to achieve a pneumatic trap for the correct bed circulation. Moreover, it could divide the flow to regulate its temperature as needed, returning directly to the hearth or passing through an exchanger that reduces its temperature (SH_2_ and RH_2_).

After the flue gas got out of the cyclone system, it transfers its energy content through a high-temperature super-heater (SH_3_), low-temperature reheater (RH_1_), the economizer (ECO), and flue gas heater (RGH) [2].

Figure 1 shows how the flue gas must pass through a gas treatment process before its evacuation through the stack. In this work, SCR and FM units were selected as abatement systems to satisfy the levels of NO_x_ and ash down to acceptable values according to current EU regulations (500 mg/Nm^3^ and 20 mg/Nm^3^, respectively, for new 500 MW_th_ facilities with solid fuel) [2].

### 2.2. Oxy-Combustion Coal-Fired CFC Supercritical Plant with CO_2_ Capture

The oxy-fuel capture system was based on the direct combustion of anthracite coal with oxygen in the absence of nitrogen. The configuration consisted of a CFB boiler, a supercritical steam cycle, and a flue gas treatment system. Both the combustion process and the heat transfer process to the thermomechanical fluid were set to be similar to the Reference Case. For that, it was necessary to control temperatures inside the hearth by a fraction of the recirculated flue gas, which reduced the excessive temperatures that could be reached because of burning with pure oxygen. The resulting flue gas of this configuration was a mixture of carbon dioxide and water vapor without nitrogen. This water vapor content could be condensed in a later stage to get a highly enriched CO_2_ stream. As a result of this stage, a stream of highly concentrated CO_2_ was obtained, which was ready for the transport stage and its definitive geological storage or to be reused for synthetic fuel production.

Finally, an oxy-combustion process requires an oxygen supply unit. Nowadays, there are different alternatives and each one is at a different development stage. According to the alternatives proposed in Portillo et al., a cryogenic air separation unit (ASU) and four configurations composed of oxygen transport membranes (OTMs) were considered for this techno-economic analysis [2]. The main characteristics are described in the following subsections.

#### 2.2.1. Case 1: Cryogenic Oxygen-Fired CFB Supercritical Plant with CO_2_ Capture

**Figure 2:**
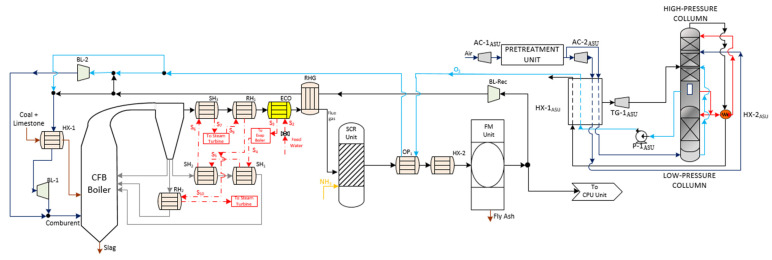
Schematic diagram of Case 1 adapted from [2,12,15].

Figure 2 displays the basic structure of Case 1 (oxygen-fired CFB process supercritical plant with CO_2_ capture). As Table 1 shows, their technical details are similar to the Reference Case concerning the type of boiler, steam cycle, and pollutant gas treatment system. The main difference to this alternative was the replacement of the comburent by oxygen supplied through an ASU technology together with recycled flue gases. As a result of this process, its combustion produced a CO_2_-rich flue gas with a water vapor molar composition of 23%. After the gas processing system, the product stream is characterized by a high purity CO_2_ stream with a water vapor molar composition of 11%.

Regarding the design of the oxygen supplier, the ASU unit was composed of a distillation column with a low-temperature multistage, and an argon column for additional oxygen purification [14]. The process started with the compression and purification of air. Cooling to cryogenic condition was reached by a heat exchanger together with after-coolers and expanders. Alternatively, the products were pressurized by small boost compressors. From a thermodynamic point of view, the power of the steam turbine increases, and the power required by the ASU and induced fan increases. As a concurrence of these energy requirements, the net power output and net power efficiency increase. Conventionally, this unit consumes about 23–47% of the total plant output, leading to an energy penalty of 7% compared with a plant without capture [2]. The oxy-combustion process requires higher investment and operational costs, resulting in energy costs in the range of 0.07 €/kWh to 10.47 €/kWh compared with conventional power plants [5].

#### 2.2.2. Oxygen Transport Membrane Applied to Oxy-Combustion Process

This work had accounted for the oxygen transport membranes (OTM) as the oxygen supplier unit for cases 2 to 5. The main reason is that this alternative could imply advantages compared with the ASU unit. According to the review published by Portillo et al. in 2019, OTM showed energy and economic savings up to 9 and 17.3%, respectively [5]. In these cases, the required oxygen in the oxy-combustion area was achieved thanks to the generation of the driving force on both sides of the membrane. The oxygen permeate rate (mol/m^2^·s) can be explained by the Wagner equation (Equation (1)) [2,5]:

(1) $$j_{O_{2}}{(\mathrm{mol}/m}^{2}{\cdot s)=C}_{\mathrm{Wagner}}\cdot \frac{T_{m}}{d_{m}}{\cdot e}^{-\frac{K_{\mathrm{Wagner}}}{T_{m}}}{\cdot \ln\;P}_{O_{2,\mathrm{ratio}\;\mathrm{avg}}}$$

where $P_{O_{2,\mathrm{ratio}\;\mathrm{avg}}}$ is the average ratio of the oxygen partial pressure between both membrane sides [bar]; C_Wagner_ and K_Wagner_ are intrinsic membrane material constants that must be determined experimentally. According to the consulted references, the available values for these constants are 1.004·10^−8^ mol/cm·s·K and 6201 K, respectively [5,16,17]; T_m_ is the absolute temperature [K]; and d_m_ is the membrane thickness [m].

As can be seen in Equation (1), the oxygen flow rate through the membrane depends on [5]:

- The Napierian logarithm of the driving force (O_2_-partial pressure ratio): the higher the difference in the partial oxygen pressure, the higher the oxygen flow rate across the membrane will be. Normally, the OTM unit achieved the oxygen flux through modes known as 4-end and 3-end (Table 2). In the 4-end concept, the difference in oxygen partial pressure is reached by a sweep stream on the permeate side coming from the oxy-combustion area. In the second mode, this driving force is accomplished by vacuum generation on the permeate side.
- Operating temperature: the higher this parameter, the higher oxygen flow rate trough the membrane is. Typically, this technology is able to withstand values of up to 1100 °C due to its physical properties [18,19].
- Thickness of the membrane: there is an inverse relationship between this variable and the oxygen flow rate. Normally, most of the material used in the OTM membrane has a thickness between 200 and 300 μm [20,21].

As well as these basic parameters, the literature recommends considering other crucial parameters as evaluation criteria. Their definition is displayed in Table 2, making a distinction between 3-end and 4-end modes.

**Table 2:** Comparative parameters for the evaluation of OTM configurations [2,5].

	Type of Mode	
	3-End	4-End
*Design*	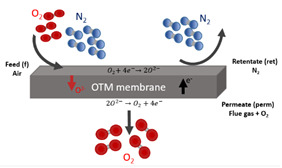	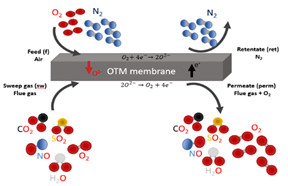
**Oxygen Separation ratio (SR)**		
*Definition*	Oxygen fraction that passes through the membrane module from the feed side to permeate side	
*Equation*	(2) $\mathrm{SR}=\frac{y_{O2,p}{\cdot m}_{\mathrm{perm}}}{y_{O2,f}{\cdot m}_{f}}$	
*Parameters*	m_perm_ and m_f_: The molar flows in the permeate side and feed side, respectively y_O2,p_ and y_O2,f_: The oxygen molar fraction in the permeate side and the feed side, respectively	
**Oxygen partial pressure ratio ($\pi_{m\;}o\;P_{O_{2,\mathrm{ratio}\;\mathrm{avg}}}$)**		
*Definition*	This parameter corresponds to the total membrane oxygen partial pressure ratio, which can be determined as the average between feed ($\pi_{f}$) and retentate ($\pi_{\mathrm{ret}}$)	
*Equation*	(3) $\pi_{m}=\frac{\pi_{f}+\pi_{\mathrm{ret}}}{2}$	
	(4) $\pi_{f}=\frac{P_{O_{2},f}}{P_{O_{2},\mathrm{perm}}}$ (5) $\pi_{\mathrm{ret}}=\frac{P_{O_{2},\mathrm{ret}}}{P_{O_{2},\mathrm{perm}}}$	(6) $\pi_{f}=\frac{P_{O_{2},f}}{P_{O_{2},\mathrm{perm}}}$ (7) $\pi_{\mathrm{ret}}=\frac{P_{O_{2},\mathrm{ret}}}{P_{O_{2},\mathrm{SW}}}$
*Parameters*	$\pi_{f},\;\pi_{\mathrm{ret}}$: Quotient of oxygen partial pressure at the feed side and the end of the membrane separation process, respectively $P_{O_{2},f},{\;P}_{O_{2},\mathrm{perm}}$, $P_{O_{2},\mathrm{ret}}$: Oxygen partial pressure in the feed, permeate, and retentate side, respectively	
**OTM system effective area (A_eff,_) [14,22]**		
*Definition*	Required area to satisfy the oxygen fraction	
*Equation*	(8) $A_{\mathrm{eff},\mathrm{Case}\;i}\;(m^{2})=j_{O_{2}}{(\mathrm{mol}/m}^{2}\cdot s)\cdot \left(y_{O2,p}\cdot m_{\mathrm{perm}}\right)$ (9) $A_{\mathrm{eff},}\;(m^{2})=N_{\mathrm{mod}}{\cdot \;A}_{\mathrm{mod}}$	
*Parameters*	A_mod_, N_mod_: Module area and module number of OTM unit to satisfy the effective area Considering membranes with a tubular design, the module geometry and calculating method had been determined through the methodology proposed by Vent et al. (2009) [14,22]	

In light of the above, this work studied four options for an OTM configuration, whose aim was to reach high oxygen differential pressure in the OTM system, as well as an economic and energy advantage compared with the conventional oxygen supplier used on a full scale. Each studied case required specific equipment for its integration into the oxy-combustion process, which implied a relevant influence during the techno-economic assessment. The main differences among cases were the heating system to achieve the temperature of the OTM unit, their location in the oxy-combustion process, and the mode to reach the oxygen flux through the membrane. Below is a brief detailed description of the four OTM alternatives proposed in this paper [2,5].

##### Oxygen-Fired CFB Supercritical Plant with CO_2_ Capture Based on OTM Unit with 3-End Mode (Case 2 and Case 3)

**Figure 3:**
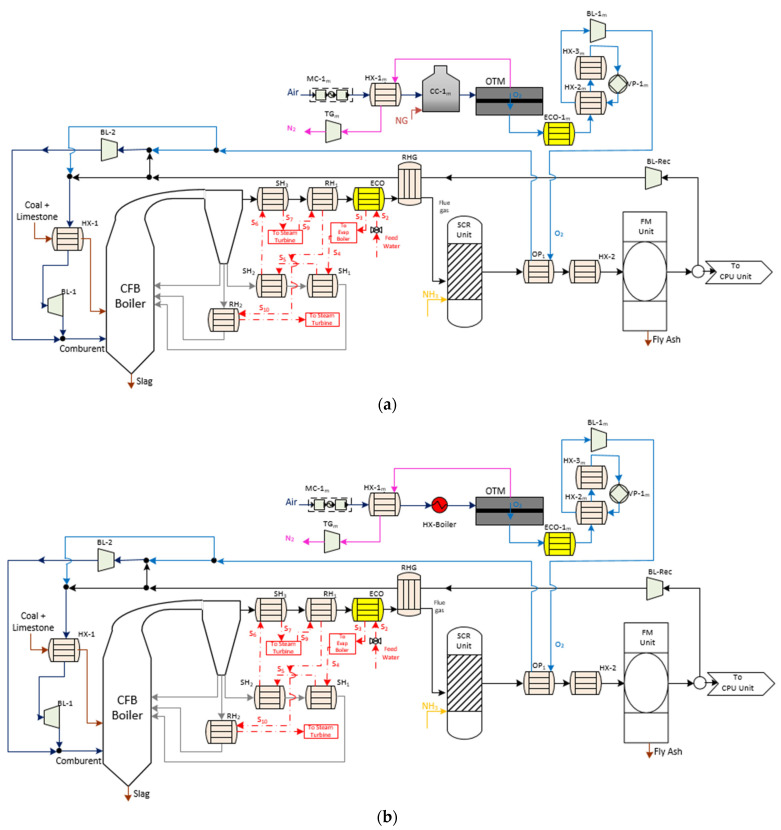
(**a**) Schematic diagram of Case 2 (OTM unit with 3-end mode and heating system with natural gas) [2,23,24] and (**b**) schematic diagram of Case 3 (OTM unit with 3-end mode and heating system with the steam cycle of the oxy-fuel process) [2,21,23].

Figure 3a displays the schematic diagram of an oxygen-fired CFB process comprising an OTM unit. In this case, the oxy-combustion area, steam cycle, and pollutant control system (SO_2_, NO_x_, and particles) maintains the same design as Case 1. Concerning the OTM unit, this case was characterized by having driven equipment, a combustion chamber, and heat exchangers. Firstly, the air feed stream was compressed by a multi-compressor (MC-1_m_) to provide high pressure on the feed side of the OTM membrane. After that, this stream passed through the heater HX-1_m_ and the combustion chamber (CC-1_m_). In the first heat exchanger, the retentate stream of the OTM membrane was responsible for giving up the heat to the air stream. Immediately, the combustion of natural gas in the combustion chamber provided the rest of the heat for the air, satisfying the required operating temperature during the air separation. This work used the same specification as done in Portillo et al. [2].

Once the air entered the membrane, the driving force was achieved by generating a vacuum within the pump VP-1_m_ (3-end mode), which was located in the permeate stream. Regarding the retentate line, the TG-1_m_ gas turbine was set after the HX-1_m_ heater to recuperate the residual energy and minimize energy requirements by MC-1_m_. This mode of energy integration was used in the rest of the OTM configuration.

At the same time, the permeate stream passed through an economizer (ECO-1_m_) and two exchangers (HX-2_m_ and HX-3_m_) before VP-1_m_. This is because of the operational limitations of VP-1_m_ in which the temperature was set at about 20 °C. One of the advantages of OTM configurations was the harnessing of energy through the TG-1_m_ turbine and ECO-1_m_ economizer, which were incorporated into the thermodynamic part of this study.

Finally, the comburent stream was mixed with the recirculation stream downstream of the treatment gasses, with its pressure and temperature conditioned by BL-1_m_ and OP.

Case 3 is shown in Figure 3b. As can be seen, both the oxy-combustion section and the auxiliary equipment of the OTM unit were similar to Case 2, except for the second exchanger used for the temperature conditioning of the feed side. In this case, another exchanger (HX-boiler), whose energy was taken from the steam extraction within the boiler of the oxy-fuel process, replaced the combustor CC-1_m_. The main goal of this change was to evaluate another type of energy integration that could affect the techno-economic assessment.

##### Oxygen-Fired CFB Supercritical Plant with CO_2_ Capture Based on OTM Unit with 4-End Mode (Case 4 and Case 5)

**Figure 4:**
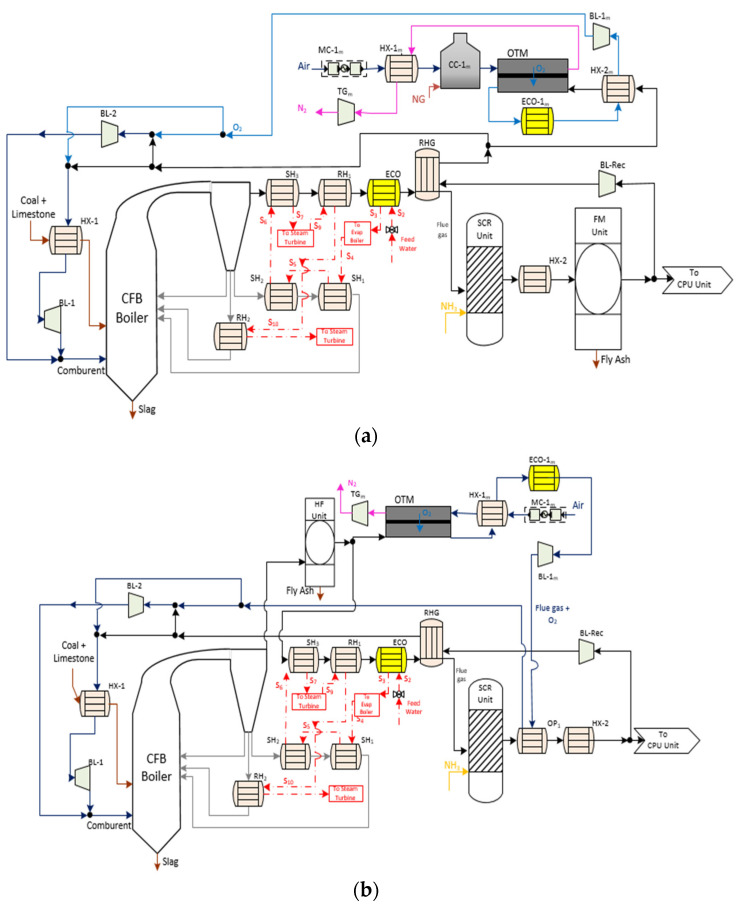
(**a**) Schematic diagram of Case 4 (OTM unit with 4-end mode and heating system with natural gas) [2,3,17] and (**b**) schematic diagram of Case 5 (OTM unit with 4-end mode; heating system with flue gas from oxy-fuel process and hot filtration) [2].

Cases 4 and 5 are displayed in Figure 4. As can be seen, these cases maintained certain similarities with previous OTM units in terms of equipment of the oxy-combustion area, steam cycle and gas treatment system.

Regarding the driving force generated by the membrane, these cases were set up according to the 4-end design. Specifically, an extraction of flue gas was the stream used to separate and sweep along the oxygen from the air stream, which was on the feed side of the membrane. The extraction point of this sweep stream from the oxy-combustion area was different in Case 4 and 5. In Case 4, the sweep gas was taken downstream of the treatment gasses after the RHG exchanger. Before passing to the membrane unit, this stream had to reach the appropriate temperature to favor a high oxygen flow rate (Figure 4a). This configuration had a gas-gas heater known as HX-2_m_, where the hot stream was the permeate stream that came out of the OTM membrane. In the counter-current direction, the airstream was subjected to the change in temperature and pressure using the same auxiliary equipment (MC-1_m_, HX-1_m_, and CC-1_m_) as Case 2. Once achieving the ideal operating conditions of the membrane, the oxygen passed from the feed stream to the permeate stream. After that, it entered the cooling system (ECO-1_m_) and HX-2_m_ as a temperature conditioning system of the impulse blower (BL-1_m_).

Case 5 (Figure 4b) was the most different configuration among the OTM alternatives. Firstly, the OTM unit was located close to the boiler area of the oxy-fuel process downstream of the hot filtration system. The reason was to take advantage of the temperature conditions of the flue gas stream to be used as the sweep gas. Moreover, this stream had to be free of particles in order to avoid clogging the pores of the material, thus reducing the possible degradation and permeability of the oxygen through the OTM membrane. According to this location, it was not necessary to include either HX-2_m_ or CC-1_m_ as in Case 4.

### 2.3. Assumptions

This techno-economic assessment considered the main assumptions and data basis proposed by Portillo et al. [2]. Regarding the operation conditions of the boiler, 105.5 kg/s of anthracite coal was affixed as coal consumption, whose ultimate analysis (% wt. dry) was composed of 52.59% carbon, 1.68% hydrogen, 2.95% oxygen, 0.88% nitrogen, 1.07% sulphur; proximate analysis 32 wt.% dry ash; 8.8 wt.%. moisture; and a lower heating value of 20,452 kJ/kg. Assuming an instantaneous coal decomposition, the products were mainly H_2_, N_2_, O_2_, H_2_O, S, and C, and the char was only made up of carbon. A supercritical boiler with a single re-heat was included, with feed water conditions of 250 °C and 317.6 bar. The boiler efficiency and electric capacity were fitted to 93.4% and 2.013 GW_th_. The operating temperature of the fluidized bed was set to 850 °C, and the temperature of the flue gas downstream boiler was 400 °C. The primary and secondary air ratio was 21 and 4%; oxygen rate was the downstream boiler value. Finally, the quantity of limestone was added to satisfy 95 and 2% of SO_2_ and SO_3_ conversion, respectively.

Regarding the steam cycle, 558 °C with 48 bar and 578 °C with 306 bar were the sub-critical and super-critical conditions, respectively. The upstream and downstream temperature and pressure of the economizer were 251 °C with 318 bar and 310 °C with 237 bar, respectively. To adjust the temperature throughout the boiler, the temperature and pressure downstream of the evaporator, SH_1_, SH_2_, SH_3_, and RH_2_ were 482 °C with 323 bar, 508.8 °C with 314 bar, 543.5 °C with 310 bar, 578.3 °C with 306 bar, and 557.2 °C with 47.4 bar, respectively. In addition, the upstream and downstream conditions of the RH_1_ were 294.6 °C with 51 bar and 410 °C with 49.2 bar, respectively. Finally, the condenser pressure and saturation temperature were 40 mbar and 29.9 °C, respectively.

For the turbine stage, this work sets the following conditions:

- High-pressure turbines with 85% isentropic efficiency: HP_1_-T from 306 to 51.02 bar, and HP_2_-T from 306 to 197.7 bar.
- Intermediate pressure turbines with 85% isentropic efficiency were IP_1_-T from 47.6 to 20.3 bar, and IP_2_-T from 20.3 to 11.4 bar.
- Low-pressure turbines with isentropic efficiency between 87 and 90%: LP_1_-T from 11.4 to 6.02 bar, LP_2_-T from 6.02 to 1.72 bar, LP_3_-T from 1.72 to 0.82 bar, LP_4_-T from 0.82 to 0.32 bar, and LP_5_-T from 0.32 to 0.04 bar.
- Pump operation conditions (isentropic efficiency): 6.06 bar (64.34%) in the condenser pump, 3.53 bar (55%) in the drain pump, 85.84 bar (83.33%) in the booster pump, and 318 bar (81.72%) in the boiler feed pump.

With respect to the concentration of flue gas contaminants, it was set at 20 ppm and 23 ppm for NO_x_ and CO, respectively, after cleaning without CO_2_ capture. In the oxy-fuel cases, their concentrations were 5 ppm and 18 ppm, respectively. Finally, the particle control with FM and HF was 99% with 150 °C and 99% with 850 °C, respectively.

In the OTM unit case, a value between 627 and 726 t/h was set as the oxygen flow to satisfy the oxy-combustion condition, depending on the established OTM configuration, and securing maximum plant efficiency. According to the oxygen percent at the inlet as well as the outlet exit of the boiler, this comburent flow was separated between the primary and secondary recirculation gas. Besides these considerations and the typical operating conditions of the OTM design, Table 3 displays the OTM input data considered during this techno-economic evaluation.

## 3. Assessment Method

**Figure 5:**
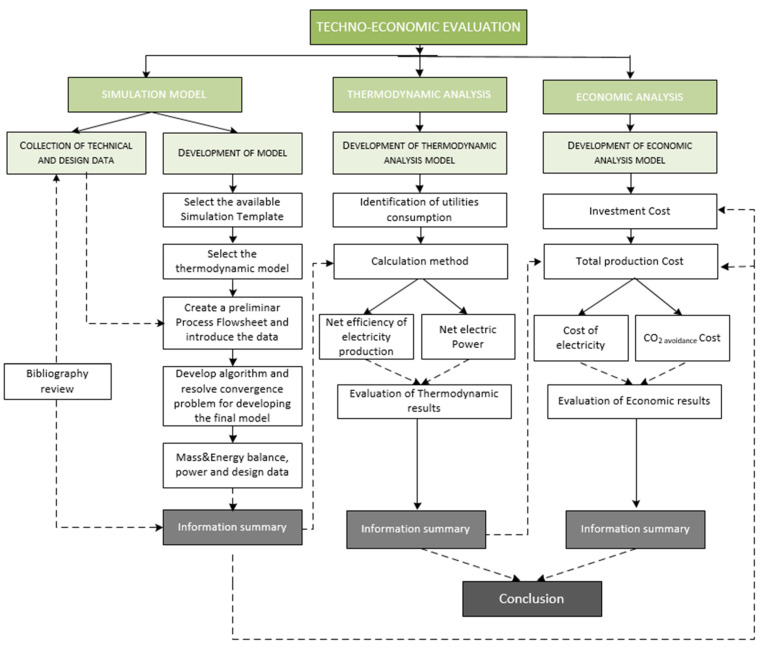
Methodology assigned to the techno-economic evaluation of this work.

For the development of the techno-economic comparison of this work, the methodology was separated into three parts: simulation model, thermodynamic analysis, and economic analysis. As can be seen in Figure 5, the method started with the development of the models, considering the flow sheets and data basis described in Section 2. Aspen Plus Dynamics was the software used to design both the base case and oxy-combustion plants. The main goal of this initial stage was to determine process variables and design data under steady-state operating conditions. Each simulation model was composed of phases, such as selecting the available simulation template, selecting the thermodynamic model, creating a preliminary process flowsheet, and introducing data.

Once the algorithms had been developed and the convergence problems solved, final models provided the relevant results concerning utility consumption and the mass and energy balances, as well as the second law of thermodynamics.

Considering the sequential steps, which are shown in Figure 5, the resolution of the thermodynamic and economic analyses for each case are described in the following sub-sections.

### 3.1. Thermodynamic Performance Assessment

In thermodynamic performance, the identification of the individual consumers was the first step. Some of the power consumptions were directly obtained from the simulations of the six configurations developed, whereas other components were obtained from the set of thermodynamic equations displayed in Table 4.

**Table 4:** Thermodynamic equations of some plant components.

Section	Equation	Refs.
Boiler & Steam cycle area	(10) $$P_{\mathrm{aux},i}\left(\mathrm{kW}\right)=P_{\mathrm{aux},\mathrm{ref}\;i}\left(\mathrm{kW}\right)\cdot {\left(\frac{{\mathrm{MW}}_{\mathrm{gross},\mathrm{ref}}}{{\mathrm{MW}}_{\mathrm{gross}}}\right)}^{\mathrm{sf}}$$ where: $P_{\mathrm{aux},\mathrm{ref}\;i}\left(\mathrm{kW}\right)$ is the auxiliary power consumption for the Reference Case study where it contemplated the auxiliaries of the boiler unit and steam cycle associated with the draft system, cooling water system, material handling, and so on $MW_{\mathrm{gross},\mathrm{ref}}$ is the Reference power output (212 MW_el,gross_). $MW_{\mathrm{gross}}$ is the power output generation fixed (863 MW_el,gross_) sf is the scaling factor (0.6)	[25,26]
Particle filtration system	(11) $P_{F,i}\left(\mathrm{kW}\right)=\frac{0.746\cdot Q\cdot \Delta P}{6356\cdot \eta }$ where: Q (ft^3^/min) is the system filtered flow rate through the modeling process The system pressure drop (ΔP) is 3.62 inches water for FM filtration system or 5.43 inches water for HF filtration system η combined fan and motor efficiency (usually 0.6 to 0.7)	[27,28,29]
SCR unit	(12) $$P_{\mathrm{SCR},i}\left(\mathrm{kW}\right)=0.150\cdot Q_{B}\cdot \left[{\mathrm{NO}}_{x,\mathrm{in}}\cdot {\;\zeta }_{{\mathrm{NO}}_{x}}+0.5\cdot \left({\Delta P}_{\mathrm{pipe}}+{\Delta P}_{\mathrm{catalyzed}}\right)\right]$$ where: $Q_{B}$ (MMBtu/h) is the boiler size The pressure increases in the pipe (ΔP_pipe_) and in catalyzed (ΔP_catalyzed_) is 2 and 0.75 inches water, respectively The NO_X_ removal efficiency (ζ_NOX_) is 0.95 ${\mathrm{NO}}_{x,\mathrm{in}}$ is determined through the modeling process	[30]
ASU unit	(13) $$P_{\mathrm{ASU}}\;\left(\mathrm{MW}\right)=3798\cdot 10^{-3}\cdot M_{O_{2}}\cdot \left[\frac{0.0736}{{\left(100-\varphi \right)}^{1.3163}}+0.8779\right]\;\mathrm{for}\;\varphi >97.5\%$$ where: φ is the O_2_ product purity (99.5 mole%) $M_{O_{2}}$ (lbmol/hr) is the total oxygen requirement from ASU	[31]
Impulse blower (BL)	(14) $P_{\mathrm{blower},i\;}\left(\mathrm{kW}\right)=\frac{0.746\cdot Q\cdot \;\Delta P}{6356\cdot \eta }$ where: Q (ft^3^/min) is the gas flow rate through the modeling process The pressure drop (ΔP) is 0.5 inches water The efficiency blower (η) is 85%	[28]
Cooling Water (HX_m_)	(15) $$P_{\mathrm{cooling}\;\mathrm{water}\;}\left(\mathrm{kW}\right)=\frac{4.7\cdot 10^{-5}\cdot M_{\mathrm{cooling}}}{1000}$$ where: $M_{\mathrm{cooling}}$ (gpm) is the cooling water flow rate	[31]
Air vacuum pump (VP)	(16) $$P_{\mathrm{VP}}\left(\mathrm{kW}\right)=23,168\cdot \dot{m_{O_{2}}\cdot }P_{\mathrm{vacuum}}^{-0.8151}$$ where: $\dot{m_{O_{2}}}$ (kg/s) is separated oxygen mass flow using the ITM unit with three-end mode (Cases 2 and 3) $P_{\mathrm{vacuum}}\;\left(\mathrm{mbar}\right)$ is the required vacuum pressure using the ITM unit with three-end mode (Cases 2 and 3)	[16]

As discussed above, the thermodynamic indicators considered in this work were the net electric power (Equation (17)), the net efficiency of electricity production (Equation (18)), and the specific energy consumption for CO_2_ capture (Equation (19)), which were given as:

(17) $$N_{\mathrm{el},\mathrm{net}}\left[{\mathrm{kW}}_{e}\right]{=N}_{\mathrm{el},\mathrm{gross}}\left[{\mathrm{kW}}_{e}\right]-{\;N}_{\mathrm{el},\mathrm{aux}}$$

where $N_{\mathrm{el},\mathrm{net}}$ is the net power available for sale to the electrical grid corresponding to each case; N_el,gross_ is the gross power minus the station service power, which is considered constant in every case studied; and $N_{\mathrm{el},\mathrm{aux}}$ is the energy penalty of the sum of the auxiliary equipment, which is required to ensure correct operation during the electric power generation in each case.

By applying Equation (18), the net efficiency of electric generation ($N_{\mathrm{el},\mathrm{net}}$) was determined as:

(18) $$\eta_{\mathrm{el},\mathrm{net}}\left(\%\right)={\;\eta }_{\mathrm{el},\mathrm{gross}\;}\left(\%\right)\cdot \frac{N_{\mathrm{el},\mathrm{net}}}{m_{\mathrm{coal}}\cdot \mathrm{LHV}}$$

where $\eta_{\mathrm{el},\mathrm{net}}$ is the net efficiency of electric generation; $\eta_{\mathrm{el},\mathrm{gross}}$ is the gross efficiency of the electric generation which is considered constant in every case studied; LHV is the low heat value of coal (kJ·kg^−1^); and m_coal_ represents the mass flow of the coal feed (kg/h).

The specific energy consumption for CO_2_ capture (SPCCC) was calculated from the formula:

(19) $$\mathrm{SPCCC}=\frac{N_{\mathrm{el},\mathrm{net}}\left[{\mathrm{kW}}_{e}\right]}{{\mathrm{kg}}_{{\mathrm{CO}}_{2,\;\mathrm{captured}\;}/h}}$$

### 3.2. Economic Performance Assessment

To carry out a realistic economic evaluation, this work considered five key parameters. On the one hand, accurate information was required about the expenses associated with the annual total capital investment (TCI_a_) and the annual total production cost (TPC_a_). On the other hand, the levelized cost of electricity (LCOE), the CO_2_ capture cost, and CO_2_ avoidance cost were considered to calculate costs related to the oxy-fuel systems. To ensure consistency in the calculation procedure, the methodology proposed by the National Energy Technology Laboratory (NETL) reports [25,32,33] was used along with various analyses on CO_2_ capture and O_2_ production technologies carried out by specialized authors [2,4,16,23,34,35,36,37]. Specifically, the TCI_a_ concept is the capital necessary for the design, construction, and start-up, which can be calculated via Equation (20):

(20) $${\mathrm{TCI}}_{a}=\frac{\mathrm{TCI}}{\frac{{\left(1+i\right)}^{n}-1}{i\cdot {\left(1+i\right)}^{n}}}$$

where TCI is the total capital investment that was determined by means of sequential calculations (Table 5) based on the sum of the equipment cost plus the auxiliary equipment for the different sections of each proposed scenario. Some of these components were estimated from the cost functions presented in Table 6. To homogenize the process, a similar investment risk technology was considered with a useful life (n) of 20 years and 7% per year interest rate (i).

**Table 5:** Methodological bases to determine TCI [28,31].

Concept	Economic Parameter	Factor
C_1_	Main equipment Cost	-
C_2_	Auxiliary equipment Cost	-
**A**	Total	C_1_ + C_2_
**B**	Purchased equipment Cost	1.18·A
C_3_	Founding Cost	0.04·B
C_4_	Handling Cost	0.5·B
C_5_	Electric system Cost	0.08·B
C_6_	Piping Cost	0.01·B
C_7_	Piping insulation Cost	0.07·B
C_8_	Painting Cost	0.04·B
**DIC**	Direct Installation Cost	0.74·B
C_9_	Engineering Cost	0.01·B
C_10_	Construction Cost	0.2·B
C_11_	Contractor’s fees	0.01·B
C_12_	Starting construction Cost	0.01·B
C_13_	Performance test	0.01·B
**IIC**	Indirect Installation Cost	0.27·B
**TCI**	Total Capital Investment	DIC + IIC

**Table 6:** The cost function of some components.

Section	Equation	Refs.
Boiler and Steam Cycle Area	(21) $$C_{\mathrm{aux},i}\left(\mathrm{MM}\$\right)=C_{\mathrm{aux},\;\mathrm{ref}\;i}\left(\mathrm{MM}\$\right)\cdot {\left(\frac{{\mathrm{MW}}_{\;\mathrm{gross},\;\mathrm{ref}}}{{\mathrm{MW}}_{\;\mathrm{gross}}}\right)}^{\mathrm{sf}}\cdot \left(\frac{{\mathrm{PCI}}_{2020}}{{\mathrm{PCI}}_{\mathrm{ref}}}\right)$$ where: $C_{\mathrm{aux},i}\left(\mathrm{MM}\$\right)$ is the installed capital cost of cost auxiliary (i) of the contemplated auxiliaries of the boiler unit and steam cycle $C_{\mathrm{aux},\mathrm{ref}\;i}\left(\mathrm{MM}\$\right)$ is the reference cost of cost auxiliary (i) of the contemplated auxiliaries of the boiler unit and steam cycle The auxiliaries contemplated in this area are shown in the flowsheets ${\mathrm{MW}}_{\mathrm{gross},\mathrm{ref}}$ is the Reference power output (212 MW_el,gross_) ${\mathrm{MW}}_{\mathrm{gross}}$ is the power output generation fixed (863 MW_el,gross_) sf is the scaling factor (0.6) Plant cost index for the year in which the capital cost is calculated (PCI_2020_ = 650) Plant cost index for the year in which the reference cost was reported (PCI_2006_ = 449.6)	[26,38,39]
**Particle filtration unit** **(FM, HF)**	(22) $$C_{\mathrm{filtration}\;\mathrm{unit}\;}\left(\$\right)=(C_{\mathrm{Fabric}\;\mathrm{filter}}+C_{\mathrm{bags}}+C_{\mathrm{auxiliry}\;\mathrm{equipment}})\cdot \frac{{\mathrm{PCI}}_{2020}}{{\mathrm{PCI}}_{\mathrm{ref}}}$$ where: C_Fabric filter_ ($) is the cost of the baghouse C_bags_ ($) is the bag cost $C_{\mathrm{auxiliry}\;\mathrm{equipment}}$ is the cost which considers the necessary auxiliaries in the unit Plant cost index for the year in which the capital cost is calculated (PCI_2020_ = 650) Plant cost index for the year in which the reference cost was reported (PCI_2002_ = 395.6)	[27,28,29]
SCR unit	(23) $$C_{\mathrm{SCR},i}\left(\$\right)=\frac{{\mathrm{PCI}}_{2020}}{{\mathrm{PCI}}_{\mathrm{ref}}}\cdot Q_{B}\cdot \left[\frac{3380\;\$}{\mathrm{MMBtu}/h}+f(h_{\mathrm{SCR}})+f\left(Q_{{\mathrm{NH}}_{3,\;\mathrm{rate}}}\right)\right]\cdot {\left(\frac{3500}{Q_{B}}\right)}^{0.35}+f\left({\mathrm{Vol}}_{\mathrm{catalyst}}\right)$$ where: $Q_{B}$ (MMBtu/hr) boilet heat input f(h_SCR_) is the adjustment for the SCR reactor height, which is calculated as: (24) $$f(h_{\mathrm{SCR}})=\left[\frac{6.12\$}{\frac{\mathrm{ft}-\mathrm{MMBtu}/h}{h}}h_{\mathrm{SCR}}\right]-\frac{187.9\$}{\mathrm{MMBtu}/h}$$ f ($Q_{{\mathrm{NH}}_{3,\;\mathrm{rate}}}$) is the adjustment for the ammonia flow rate, which is determinated as: (25) $$Q_{{\mathrm{NH}}_{3,\;\mathrm{rate}}}=\left[\frac{411\$}{\frac{\mathrm{lb}}{h}}\cdot \frac{{\;\dot{m}}_{\mathrm{reag}}}{Q_{B}}\right]-\frac{47.3\$}{\left(\mathrm{MMBtu}/h\right)}$$ $f\left({\mathrm{Vol}}_{\mathrm{catalyst}}\right)$ is the capital cost for the initial charge of the catalyst, which is calculated as: (26) $${\mathrm{Vol}}_{\mathrm{catalyst}}={\mathrm{Vol}}_{\mathrm{catalyst}}\cdot {\mathrm{CC}}_{\mathrm{initial}}$$ where ${\mathrm{Vol}}_{\mathrm{catalyst}}$ is in ft^3^ and CC initial is the cost of the initial catalyst ($/ft^3^) for a ceramic honeycomb catalyst. Plant cost index for the year in which the capital cost is calculated (PCI_2020_ = 650) Plant cost index for the year in which the reference cost was reported (PCI_2002_ = 395.6)	[40]
OTM membrane	(27) $$C_{\mathrm{OTM}}\left(\$\right)=C_{\mathrm{ref}}^{o}\cdot \frac{m_{O_{2}}}{J_{O_{2}}}\;\cdot \frac{{\mathrm{PCI}}_{2020}}{{\mathrm{PCI}}_{\mathrm{ref}}}$$ where: $C_{\mathrm{ref}}^{o}$ is the purchased reference cost base equal to 75 $/m^2^ $m_{O_{2}}$ (mol/s) is separated oxygen mass flow using the ITM unit $J_{O_{2}}$ (mol/m^2^·s) is the oxygen permeate rate, which was determined by Equation (1) Plant cost index for the year in which the capital cost is calculated (PCI_2020_ = 650) Plant cost index for the year in which the reference cost was reported (PCI_2009_ = 511.8)	[41]
ASU unit	(28) $$C_{\mathrm{ASU}}\;\left(\mathrm{MM}\$\right)=\frac{14.35\cdot N_{t}\cdot T_{a}^{0.067}}{1000\cdot {\left(1-\varphi \right)}^{0.073}}\cdot {\left(\frac{M_{O_{2}}}{N_{o}}\right)}^{0.852}\cdot \left(\frac{{\mathrm{PCI}}_{2020}}{{\mathrm{PCI}}_{\mathrm{ref}}}\right)$$ where: T_a_ is the ambient air temperature (°F). A T_a_ equal to 95 °C was chosen to meet the condition 20 °F < Ta < 95 °F N_t_ is the total number of production trains. The condition to be met is the maximum train capacity of 11,350 lbmol/h) N_o_ is the number of operating production trains. The condition to be met is 625 < $\frac{M_{O_{2}}}{N_{o}}$ < 11,350 lbmol/ $M_{O_{2}}$ (lbmol/hr) is the total oxygen requirement of the ASU φ is the O_2_ product purity (mole%) = 99.5 Plant cost index for the year in which the capital cost is calculated (PCI_2020_ = 650) Plant cost index for the year in which the reference cost was reported (PCI_1989_ = 355.4)	[31]
Combustor Chamber (CC-1_m_)	(29) $$C_{\mathrm{CC}-1_{m}}=10^{\left(K_{1}+K_{2}\cdot \log(P_{\mathrm{cc}-1_{m}})+K_{3}\cdot {\left[\log\left(P_{\mathrm{cc}-1_{m}}\right)\right]}^{2}\right)}\cdot F_{p}\cdot \frac{{\mathrm{PCI}}_{2020}}{{\mathrm{PCI}}_{\mathrm{ref}}}$$ F_p_ = Pressure cost factor (1) $P_{\mathrm{CC}-1_{m}}$ (kW) is the power produced by the equipment K_i_ is the characteristic calculation parameters for equipment i with a value equal to 7.349, −1.167, and 0.203, respectively Plant cost index for the year in which the capital cost is calculated (PCI_2020_ = 650) Plant cost index for the year in which the reference cost was reported (PCI_2006_ = 499.6)	[42]
Air Economizer (ECO-1_m_)	(30) $$C_{\mathrm{ECO}-1_{m}}\left(\$\right)=C_{\mathrm{HX},i}^{o}\cdot F_{\mathrm{BM}}\cdot F_{p}\cdot F_{s}\cdot \frac{{\mathrm{PCI}}_{2020}}{{\mathrm{PCI}}_{\mathrm{ref}}}$$ where: F_BM_ = Material cost factor (2.9) F_p_ = Pressure cost factor (1) F_s_ = Piping and control cost factor (1.7) $C_{\mathrm{ECO}\_1m}^{o}$ is the equipment cost for ambient pressure using carbon steel, which was fitted to the following equation: (31) $$\log_{10}C_{\mathrm{ECO}\_1m}^{o}=K_{1}+K_{2}\log_{10}\left(A\right)+K_{3}{\left[\log_{10}\left(A\right)\right]}^{2}$$ where: A is the heat transfer surface area for the equipment i (m^2^) K_i_ is the characteristic calculation parameters for the equipment i with a value equal to 4, 0.3698, and 0.0025, respectively Plant cost index for the year in which the capital cost is being calculated (PCI_2020_ = 650) Plant cost index for the year in which the reference cost was reported (PCI_2009_ = 511.8)	[42]
Heat exchanger (RGH, HX, OP-1)	(32) $$C_{\mathrm{HX},i}\left(\$\right)=\left(B_{1}+B_{2}\cdot F_{M}\cdot F_{p}\right)\cdot C_{\mathrm{HX},i}^{o}\cdot F_{s}\cdot \frac{{\mathrm{PCI}}_{2020}}{{\mathrm{PCI}}_{\mathrm{ref}}}$$ where: B_i_ is constants with the value equal to 0.96 and 1.21, respectively F_M_ = Material cost factor (2.9) F_p_ = Pressure cost factor (1) F_s_ = Piping and control cost factor (1.7) $C_{\mathrm{HX}}^{o}$ is the equipment cost for ambient pressure using carbon steel which was fitted with Equation (30) where K_i_ is the characteristic calculation parameters for equipment i with a value equal to 4, −0.23, and 0.05, respectively Plant cost index for the year in which the capital cost is calculated (PCI_2020_ = 650) Plant cost index for the year in which the reference cost was reported (PCI_2006_ = 499.6)	[42,43]
Impulse blower (BL)	(33) $$C_{\mathrm{blower},\;i\;}\left(\$\right)=\left(C_{\mathrm{BL},i}^{o}\cdot F_{\mathrm{BM}}\cdot F_{s}\right)\cdot \frac{{\mathrm{PCI}}_{2020}}{{\mathrm{PCI}}_{\mathrm{ref}}}$$ where: F_s_ = Piping and control cost factor (2) F_BM_ = bare module equipment cost (2.8) $C_{\mathrm{BL},i}^{o}$ is the purchased cost base conditions for ambient pressure using carbon steel, which was fitted to the following equation: (34) $$C_{\mathrm{BL},i}^{o}=\{\begin{array}{l} \frac{M_{i}}{100}\cdot 10^{R_{1}+R_{2}\log_{10}\left(100\right)+K_{3}{\left[\log_{10}\left(100\right)\right]}^{2}{\;\;\;\mathrm{if}\;M}_{i}\;\geq \;100\frac{{\;m}^{3}}{s}}\\ 10^{R_{1}+R_{2}\log_{10}\left(100\right)+K_{3}{\left[\log_{10}\left(100\right)\right]}^{2}{\;\;\;\;\;\;\;\;\;\;\;\;\;\;\;\mathrm{if}\;M}_{i}<100\;\frac{m^{3}}{s}} \end{array}$$ where: $M_{i}$(m^3^/s) is the gas flow determined in the modeling process for the equipment i R_i_ is the characteristic calculation parameters for equipment i with a value equal to 4, -0.35, and 0.45, respectively Plant cost index for the year in which the capital cost is calculated (PCI_2020_ = 650) Plant cost index for the year in which the reference cost was reported (PCI_2009_ = 511.8)	[28]
Air vacuum pump (VP-1_m_)	(35) $$C_{\mathrm{VP}-1m}\left(\$\right)=4200\cdot {\left(60\cdot m_{O_{2}}\cdot \frac{T_{\mathrm{in}}}{P_{\mathrm{in}}}\right)}^{0.55}\cdot \frac{{\mathrm{PCI}}_{2020}}{{\mathrm{PCI}}_{\mathrm{ref}}}$$ where: $m_{O_{2}}$ (kmol/s) is separated oxygen mass flow using the ITM unit with three-end mode (Cases 2 and 3) Plant cost index for the year in which the capital cost is calculated (PCI_2020_ = 650) Plant cost index for the year in which the reference cost was reported (PCI_2003_ = 401.7) P_in_ and T_in_ are the inlet pressure (kPa) and temperature (K) of the equipment, respectively	[44]
Multicompressor (MC-1_m_)	(36) $$C_{\mathrm{MC}-1_{m}}\left(\$\right)={\left(7900\cdot {\mathrm{HP}}_{\mathrm{ref}}\right)}^{0.62}\cdot {\left(\frac{{\mathrm{HP}}_{\mathrm{base}}}{{\mathrm{HP}}_{\mathrm{ref}}}\right)}^{\mathrm{sf}}\cdot \frac{{\mathrm{PCI}}_{2020}}{{\mathrm{PCI}}_{\mathrm{ref}}}$$ where: $C_{\mathrm{MC}-1_{m}\;}\left(\$\right)$ is the installed capital cost of the multicompressor used in OTM units ${\mathrm{HP}}_{\mathrm{base}}$ (HP) is the power consumed by the designed equipment ${\mathrm{HP}}_{\mathrm{ref}}$ (HP) is the power consumed by the reference equipment sf is the scaling factor (0.77) Plant cost index for the year in which the capital cost is calculated (PCI_2020_ = 650) Plant cost index for the year in which the reference cost was reported (PCI_2005_ = 468.2)	[42]
Air Turbine (TG_m_)	(37) $$C_{\mathrm{TGm}}\left(\$\right)=\left(3644.3\cdot P_{{\mathrm{TG}}_{m}}^{0.7}-61.3\cdot P_{{\mathrm{TG}}_{m}}^{0.95}\right)\cdot \frac{{\mathrm{PCI}}_{2020}}{{\mathrm{PCI}}_{\mathrm{ref}}}$$ where: Plant cost index for the year in which the capital cost is calculated (PCI_2020_ = 650) Plant cost index for the year in which the reference cost was reported (PCI_2008_ = 575.4) C_TGm_ ($) is the installed capital cost of the air turbine used in OTM units $P_{{\mathrm{TG}}_{m}}$ (kW) is the power produced by the gas turbine	[45,46]

Regarding the total production cost (TPC), it is necessary to estimate direct annual costs (DAC) and indirect annual costs (IAC) [28]. The first concept included variable direct annual costs (raw materials; utilities such as steam, electricity, fuel, cooling water; and waste treatment and disposal) and semi-variable direct annual costs (Operating, supervisory, maintenance) and replacement parts. In the case of the direct variable costs, this was calculated in this study as shown in Equation (38):

(38) $${\mathrm{AC}}_{\mathrm{variable},\mathrm{ix}}={\;q}_{\mathrm{variable},\mathrm{ix}}\cdot C_{\mathrm{variable},\mathrm{ix}}\cdot \mathrm{CF}$$

where:

- AC_variable,ix_ is the annual cost for each variable concept considered in each case.
- q_variable,ix_ is the make-up variable concept consumption rate considered in each case.
- C_variable,ix_ is the unit cost of each variable concept considered in each case.
- CF is the capacity factor (0.85).

Concerning the semi-variable direct annual cost, it must be pointed out that the calculation criteria applied was more or less strict depending on whether the technology was consolidated or not. In this sense, the areas such as boiler, steam cycle, SCR unit, and FM unit were strengthened technology, so similar criteria to EPA [28] were applied. On the other hand, for second-generation technologies, such as the OTM unit or the hot filtration unit, strict criteria were applied because both technologies would be more complex to handle and operate.

Regarding the IAC concept, this work considered aspects such as property taxes, insurance, general and administrative (G&A), and capital recovery costs. These items were calculated following the method proposal by EPA 2002 [28].

To complete the economic comparison between the selected cases in this work, the following key indicators were considered:

- LCOE: Cost of electricity expressed in current dollars (2020) per net megawatt hour, which was calculated using Equation (39) [39,47,48]:

(39) $$\mathrm{LCOE}\;[M\$/\mathrm{MWh}]=\frac{\left({\mathrm{TCI}}_{a}\right)+(\mathrm{DAC})+(\mathrm{IAC})}{{\mathrm{CF}\cdot N}_{\mathrm{el},\mathrm{net}}\cdot 360\cdot 24}$$

where the numerator represents the sum of the annual investment costs and all annual fixed and variable costs (M $/y); the $N_{\mathrm{el},\mathrm{net}}$ (MW) is the net power available for sale to the electrical grid corresponding to each case; and CF is the capacity factor (0.85).

- CO_2_ capture cost (C_cap_) and CO_2_ avoidance cost (C_av_): Key parameters were calculated with the following equations [49,50,51]:
  (40) $$C_{\mathrm{cap}}[\$/\mathrm{ton}]=\frac{{(\mathrm{LCOE}}_{\mathrm{capture}}{-\mathrm{LCOE}}_{\mathrm{no}\;\mathrm{capture}})\;[\$/\mathrm{MWh}]}{\mathrm{CO}2\;\mathrm{captured}\;[\mathrm{tn}/\mathrm{MWh}]}$$

Equation (40) expresses the relationship between the difference in costs between alternatives with capture versus the Reference Case without capture and the specific tonne of CO_2_ captured. The CO_2_ balance captured was obtained by the simulation stage:

(41) $$C_{\mathrm{av}}{[\$/\mathrm{ton}]=C}_{\mathrm{cap}}\cdot \frac{\mathrm{CC}\;}{(\eta_{\mathrm{CAC}}{/\eta }_{\mathrm{wCAC}})-\;(1-\mathrm{CC})}$$

where C_av_ and C_cap_ are the CO_2_ avoidance and capturing costs, respectively; CC is the fraction of CO_2_ capture; and η_CAC_ and η_WCAC_ are the net efficiency of electric generation of the plant with/without capture.

This equation collects the quotient between the difference in costs between alternatives with/without capture and the specific tonne of CO_2_ (per electrical net megawatt hour produced) that are not emitted with the implementation of each technology [52].

## 4. Results and Discussion

The process and economic technical capability of the six coal-based CFB power plants cases were assessed based on the simulation models developed in the commercial software Aspen Plus. In this section, an analysis of the performance of the proposed processes was presented, focusing on the net electric power, the net efficiency of electricity production, and the specific energy consumption for CO_2_ capture. In addition, TCI_a_ and TPC_a_ were calculated to evaluate the effects of the price of electricity with key parameters such as LCOE, the CO_2_ capture cost, and CO_2_ avoidance cost.

**Figure 6:**
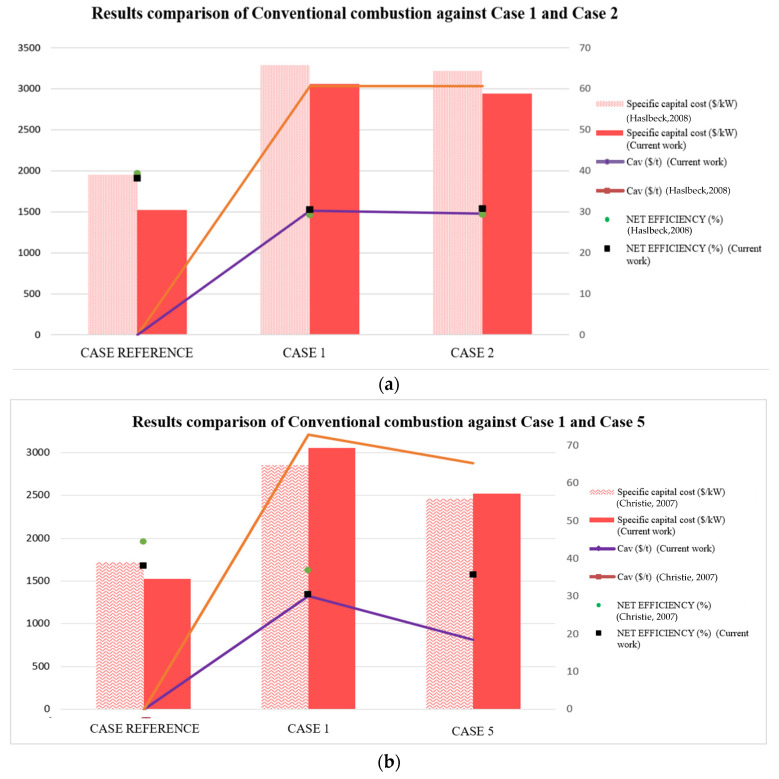
(**a**) Comparison among consulted literature (Haslbeck, 2088 [25]) and models developed in this work, considering Case reference, Case 1, and Case 2. (**b**) Comparison among consulted literature (Christie, 2007 [39]) and models developed in this work, considering Case reference, Case 1, and Case 5.

To begin with, the model verification was carried out through a comparison of the present results with the techno-economic aspect available in the literature. Despite the industrial relevance for the development of OTM units as an oxygen supplier to minimize the energy and economic limitations of other technologies, the cases proposed could not be validated in full since there was not an exactly similar system in the literature. Thus, four cases were considered for the validation of the models, distinguishing between a conventional combustion against the oxy-combustion process with ASU technology, 4-end design, or 3-end design. The net efficiency (%), specific capital cost ($/kW_el,net_), and C_av_ ($/t CO_2_ avoidance) were the comparative parameters selected in the validation model, as can be seen in Figure 6. All data were updated to 2020, which was the reference year of this work.

As shown in Figure 6, both the consulted literature data and this current work data were in good agreement without showing significant variations. Comparing the net efficiency in Case Reference, the standard deviation obtained between our model and the data reported by Ref 1 and Ref 2 was less than 3%. Moreover, a decrease in net efficiency was observed when a CO_2_ capture was included in the coal-based CFBC power plant. This tendency was observed both in the literature (Ref 1 [25], Ref 2 [38]) and in this current work. The maximum net plant efficiency drop was close to 10.1 points with the ASU technology used as an oxygen supplier, with a standard deviation of 1.2% between the references and the current work. Using the OTM as the oxygen supplier, the average drop was less than 7.7 points between the references consulted and this current work, with their standard deviations equal to 1.4 and 1.5 for Cases 2 and 5, respectively.

Considering the specific capital cost, the standard deviation between the consulted literature and the Reference Case of this work was equal to 176 $/kW_el,net_. In the case of ASU technology (Case 1) as the oxygen provider, the standard deviation was 179 $/kW_el,net_, while OTM technology entailed standard deviations close to values between 29 $/kW_el,net_ and 138 $/kW_el,net_, depending on a four-end or three-end design. Concerning the C_av_ ($/t CO_2_ avoidance), the average standard deviation approached the value between 18 and 23 $/t CO_2_ avoidance, depending on the oxygen supplier selected.

### 4.1. Process Performance Comparison

Table 7 summarizes the main performance of the six cases, with net electric power, net efficiency of electricity production, and the breakdown of auxiliary power consumption as the comparative parameters considered in this work. In addition to these parameters, environmental and design aspects were weighed in this comparison process.

In all cases, a gross power output of 863 MW_el,gross_ was set, which required the same coal flowrate (105.5 kg/s). Obviously, the implementation of a CO_2_ capture system in a power generation plant implied a decrease in net power output and net efficiency. In this sense, the additional energy requirement of the equipment load ranged from 52 to 180 MW_el,net_. In Table 7, the analysis of the distribution of the auxiliary power consumption is presented, where the oxygen supplier unit is the primary source of the efficiency penalty, either with an OTM unit or ASU technology. In Case 1, the electricity consumption decreased to 660.196 MW_el,net_ due to the presence of ASU technologies as the oxygen supplier, which entailed a net efficiency drop of 7.6% compared with the Reference Case (conventional process without CO_2_ capture). This energy penalty is close to results reported in the literature [8,53,54]. Taking into account the OTM units, Cases 5 (OTM unit with 4-end mode, heating system with flue gas from oxy-fuel process and hot filtration) and 2 (OTM unit with 3-end mode and heating system with natural gas) were the configurations with lower efficiency penalty, at 771.122 MW_el,net_ and 664.528 MW_el,net_, respectively. Compared with the Reference Case, the efficiency drops in both cases were equal to 2.43 and 7.37%, respectively. It is worth highlighting the fact that a hot filtration unit as the particulate matter abatement system, which was chosen in Case 5, entailed an energy saving (2.43%) with respect to FM as it did not require thermal conditioning of the flue gas stream before passing to the membrane unit.

From an environmental point of view, the results showed insignificant variations. The CO_2_ emissions for oxy-fuel cases were fewer than 130 g/kW_el,net_·h, reaching a value of 0 for Cases 1, 3, and 5. These results correlated well with the consulted literature [9,54,55], showing a standard deviation of 21 among its values. As for specific energy consumption for CO_2_ capture (SPCCC), the average value was equal to 0.9 kW_el,net_·h/kg_CO2 captured_ with a standard deviation of 0.06. In this regard, Case 5 could be the best option, showing 1.01 kW_el,net_·h/kg_CO2 captured_ with 100% CO_2_ recovery.

Regarding the design aspects in OTM configurations, cases with 3-end design entailed smaller membrane areas, as well as specific membrane areas (m^2^/kW_el,net_). Cases 2 and 3 required membrane areas of 413,000 m^2^ and 409,000 m^2^ with 0.62 m^2^/kW_el,net_ and 0.64 m^2^/kW_el,net_ of specific membrane area, respectively. The main reason for these results is that both cases operated at higher temperatures, thus providing a higher oxygen flow under the same operating conditions. Compared with the literature data [3,10,16,17,38], these results were in good agreement, with slight differences among them.

### 4.2. Economic Performance Comparison

Table 8 summarizes the economic comparison of selected configurations through the economic model described in Section 3.2. As expected, oxy-fuel configurations implied an extra cost compared with conventional ones without CCS configuration (Reference Case) as a consequence of avoiding CO_2_ emissions into the atmosphere as reported for oxy-fuel combustion in the industry [8]. The Reference Case presented a TCI of 1250 M$, whereas the rest of the cases required increases of between 38 and 27% in 2020. Toan et al. and Maas et al. reported a growth of 22 and 28%, respectively, in a similar economic performance to this work [8,9]. As can be seen in Table 8, OTM configurations (Cases 2, 3, 4, and 5) showed the lowest TCI compared with that of Case 1, supposing economic savings between 3 and 15%. Comparing three-end and four-end designs, Cases 3 and 4 were the configurations with the lowest values for TCI (1708 M$ and 1845 M$, respectively, in 2020). The specific capital costs ($/kW _el,net_) of Cases 2 and 4 were higher those of Cases 3 and 5, which had a larger net electricity production.

**Figure 7:**
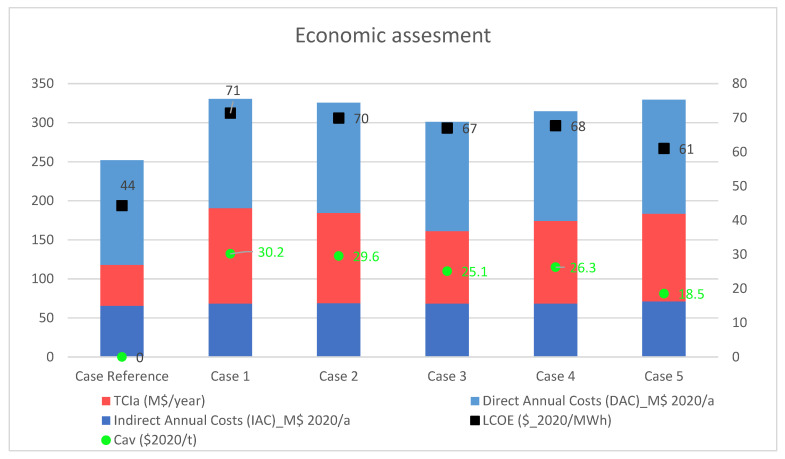
Economic assumption of CFB power plant with and without carbon capture.

In terms of total cost production, the contribution of TCI_a_, DAC, and IAC of each power plant are shown in Figure 7, where the order of the figure legend follows that of the bar chart. Oxy-fuel configurations showed a slight difference in the DAC values with a standard deviation of 2.3 M$/y, with Case 5 being the option with the highest direct annual cost. This extra cost could be a consequence of using the hot filter unit instead of a fabric filter like the rest of the configurations.

Figure 7 presents a comparison of the LCOE and mitigation of CO_2_ using an 85% capacity factor. The LCOE of Case Reference was 44 $/MW _el,net_ h, which is comparable to that of a USC coal-fired power plant (44.6 $/MW _el,net_ h) as studied by Toan et al. [8]. Comparing the LCOE between oxy-fuel alternatives, Case 1 showed up to 14% more than Case 5 (71 $/ MW _el,net_ h vs 61 $/MW _el,net_ h). In the 3-end configurations, Case 4 presented more favorable results with 67 $/MW _el,net_ h of LCOE at 6% lower than Case 1. Overall, it was reported in the literature that the LCOE of oxy-coal combustion was between 66 $/MW _el,net_ h and 72 $/ MW _el,net_ h for ASU technology [8,54,56], and 63.48 $/ MW _el,net_ h [56] and 67.30 $/MW _el,net_ h [57] for OTM cases.

Concerning the cost impact associated with CO_2_ mitigation, the CO_2_ capture cost (C_cap_) and the CO_2_ avoidance cost (C_av_) are shown in Table 8 and Figure 7. Case 1 (cryogenic-based plant) showed 23.1 $/t_CO2captured_ and 30.2 $/t_CO2_ avoidance, and is in line with the results reported in the literature [8,50]. Compared with the OTM-based plant, Case 1 implied an increase between 5–28% and 2–39%, which is within the range reported in the literature [38,57]. Therefore, this work has shown that the integration of the OTM unit into the oxy-combustion process also showed a profit regarding CO_2_ mitigation. Therefore, Case 5 again represented the most advantageous alternative with 18.5 $/tCO_2_ avoidance.

According to these results, it was confirmed that Case 5 (OTM unit with 4-end mode with hot filtration and heating system through flue gas from oxy-fuel process) is a more promising CCS technology owing to the higher electricity efficiency and lower LCOE than every alternative proposed with carbon capture.

## 5. Conclusions

In principle, coal-based power generation continues to be one of the main contributors of CO_2_ emissions as a consequence of the energy demand by society. To assure a future energy system that can be highly efficient and eco-friendly, this work analyzed the impact of CCS technology on an 863 MW conventional coal-fired power plant through a techno-economic analysis. As key assessment indicators, this paper considered the net electric power, the net efficiency of electricity production, and the specific energy consumption for CO_2_ capture, as well as capital investment (TCI), total production cost (TPC), levelized cost of electricity (LCOE), and cost of CO_2_ capture and CO_2_ avoidance.

Under these premises, this work developed one wide variety of five scenarios which led us to identify the economic feasibility of operating the oxy-fuel combustion based on two oxygen supplier types, two options of participle filtration, and several options of location and the energy integration. These cases were compared under the same assumptions and data basis, including coal feed rate, gross power output, and temperature and pressure of captured CO_2_. Herein, our study indicates a strong impact during the CO_2_ capture process in a power plant on both the thermodynamic and economic assessment.

In terms of the net power output, Case 1 (cryogenic oxygen-fired CFB supercritical plant) showed a lower value than 823.493 MW _el,net_, which was exhibited by the Reference Case. In this sense, its efficiency drop was 7.4%. Although membrane-based oxygen-fired CFB supercritical plants also displayed an energy penalty, this work demonstrated that Cases 2, 4, and 5 involved less energy using OTM units as the oxygen supplier instead of ASU technology. In fact, the optimal performance is achieved by Case 5 as its efficiency drop was substantially below the energy requirement shown by Case 1 (2.4 vs. 7.6%). In addition, the thermodynamic performance analysis revealed that the four-end design with hot filtration supposed an energy savings compared with a three-end design with particulate filtration based on fabric filter. Regarding the specific energy consumption for CO_2_ capture, this work predicted negligible variations, showing an average value of 0.9 kW _el,net_ ·h/kg. On this matter, Case 5 was again the best option as its SPCCC was equal to 1.01 kW _el,net_ ·h/kg CO_2_ capture with 100% of CO_2_ recovery.

The economic analysis revealed that the energy generation costs depended greatly on the individual boundary conditions, the energy integration system, and the equipment considered in each case. Concerning the TCI, a CCS unit implied an extra investment compared with the Reference Case. Cases 3 and 5 evidenced the potential of OTM units as oxygen suppliers in the power station plant since their economic savings were 402 $/kW_el,net_ and 536 $/kW_el,net_ with respect to Case 1. In terms of total cost production, Case Reference displayed 267 M$/y in 2020, whereas the oxy-fuel cases required between 316 M$/y and 346 M$/y. The TPC of Case 1 was the highest, which means that cryogenic technology not only needs more investment but its start-up costs are also more expensive than OTM technology. Comparing OTM technologies, the lowest TPC were in Cases 3 and 4. Therefore, the results showed that there was not a clear winner with respect to the type of design (3-end vs. 4-end design) without considering its integration into the oxy-combustion process and its heating system. Furthermore, the results showed that Case 5 would result in the most cost-effective oxy-combustion coal-fired technology, with 61 $/MW _el,net_ h of LCOE and a CO_2_ avoidance cost of 18.5$/t. This entailed an economic savings of 14 and 38.7%, respectively, compared with Case 1. In summary, it can be said that membrane-based CCS power plants represent an attractive option from a techno-economic point of view. Through the combination of oxy-fuel combustion with hot filtration and an OTM unit with a 4-end design, the most attractive configuration could be determined to encourage its development in the field as a carbon capture alternative. However, the process is far from being commercially mature and still requires intensive research concerning the development of the design, materials, and scale of both the OTM unit and the hot filtration unit.

## Figures and Tables

**Table 1:** Technical characterization of the plants assessed adapted from [2].

Cases	Characteristic							
	Boiler	Combustion Type	Treatment Gases			Characteristic ITM Unit		
			De-SO_x_	De-NO_x_	Filtration System	* Driving Force	** Heating System	*** Location into Oxy-Combustion
Reference	CFB	Conventional	Into boiler	SCR	FM	-	-	-
1	CFB	Cryogenic oxygen-fired	Into boiler	SCR	FM	-	-	DTG
2	CFB	Membrane-based oxygen-fired	Into boiler	SCR	FM	3-end mode	Combustor with natural gas	DTG
3	CFB	Membrane-based oxygen-fired	Into boiler	SCR	FM	3-end mode	Heat exchange with steam cycle from the oxy-fuel process	DTG
4	CFB	Membrane-based oxygen-fired	Into boiler	SCR	FM	4-end mode	Combustor with natural gas	DTG
5	CFB	Membrane-based oxygen-fired	Into boiler	SCR	HF	4-end mode	Heat exchange with flue gas from the oxy-fuel process	DHF

* Type of driving force across the membrane using vacuum (3-end mode) or sweep gas from flue gas (4-end mode). ** Heating system necessary to achieve air separation across the membrane unit. *** Location: DTG (Downstream of Treatment Gasses) DHF (Downstream of Hot Filter).

**Table 3:** Main assumptions for OTM units [2].

**Case**	**OTM Unit**					
	$\pi_{\mathrm{Mem}}$	**SR**	**Feed Side (Temperature, Pressure)**		**Retentate Side (Temperature Conditions, ΔP)**	
**2**	**3**	70%	850 °C, 15 bar		Isotherm conditions, 0.05 bar	
**3**	3	70%	850 °C, 15 bar		Isotherm conditions, 0.05 bar	
**4**	10.5	70%	m_feed_ = 3.6·m_FG_ (kg/s), T_feed_ = T_FG_ + 200 °C, 15 bar		Isotherm conditions, 0.23 bar	
**5**	10.5	70%	m_feed_=1.3·m_FG_ (kg/s), T_feed_ = T_FG_ − 100 °C, 15 bar		Isotherm conditions, 0.23 bar	
**Case**	**Multicompressor (MC-1_m_)**					
	**Outlet pressure**	**Stages numbers**	**Isentropic efficiency**	**Mechanical efficiency**	**Refrigeration system**	
**2**	15 ÷ 15.5 bar	2	82%	90%	40%·W_compressor_	
**3**					40%·W_compressor_	
**4**					40%·W_compressor_	
**5**					30%·W_compressor_	
**Case**	**Air Turbine (TG_m_)**			**Pressure mechanical devices (BL-1_m_)**		
	**Outlet pressure**	**Isentropic efficiency**	**Mechanical efficiency**	**Outlet pressure**	**Isentropic efficiency**	**Mechanical efficiency**
**2**	1 bar	85%	98%	1.12 bar	85%	90%
**3**						
**4**						
**5**						
**Case**	**HX-1_m_**		**HX-2_m_**		**HX-3_m_**	
	**ΔP**	**Cold stream outlet temperature**	**ΔP**	**Cold stream outlet temperature**	**ΔP**	**Hot stream outlet temperature**
**2**	3%·inlet pressure (bar)	600 °C	3% inlet pressure (bar)	88 °C	3% inlet pressure (bar)	20 °C
**3**		660 °C		88 °C		
**4**		600 °C		575 °C		
**5**		725 °C		-		
	**Combustor Chamber (CC-1_m_)**		**OP**		**Air Economizer (ECO-1_m_)**	
**Case**	**Outlet temperature**	**Loss power**	**ΔP**		**Cold stream outlet temperature**	**Hot stream outlet temperature**
**2**	850 °C	10%·Q_inlet_ KW	3%·inlet pressure (bar)		350 °C	320 °C
**3**	-	-				
**4**	850 °C	10%·Q_inlet_ KW	-		-	
**5**	-	-	3% inlet pressure (bar)		350 °C	

**Table 7:** Main technical parameters of the studied power plants with and without carbon capture.

Main Plant Data	Reference Case	Case 1	Case 2	Case 3	Case 4	Case 5
Coal flowrate (kg/s)	105.5	105.5	105.5	105.5	105.5	105.5
Coal lower heating value (MJ/kg)	20.45	20.45	20.45	20.45	20.45	20.45
Gross power output (MW_el,gross_)	863	863	863	863	863	863
Gross power efficiency (%)	38.4	38.4	38.4	38.4	38.4	38.4
Combustion area & Steam cycle (MW_el_)	36.524	25.574	23.419	23.149	23.435	22.733
Particles unit control (MW_el_)	0.937	1.087	1.087	1.087	0.798	5.611
De-NOx (MW_e_)	2.296	2.021	2.021	2.027	2.084	2.020
Cryogenic ASU load (MW_el_)	-	174.372	-	-	-	-
OTM unit (MW_e_)	-	-	172.195	193.420	172.885	61.764
Total equipment load (MW_el_)	39.757	203.054	198.722	219.683	199.202	92.128
Net power output (MW_el,net_)	823.493	660.196	664.528	643.567	664.048	771.122
Net efficiency (%)	38.2	30.6	30.8	29.8	30.8	35.7
Efficiency drop (%-points)	-	7.57	7.37	8.34	7.39	2.43
Carbon capture rate (%)		100	89.8	100	93.5	100
CO_2_ capture rate (kg/s)	0	208.5	209.0	209.0	211.2	211.3
SPCCC (kW_el,net_ h/kg CO_2_ captured)	-	0.88	0.88	0.86	0.87	1.01
CO_2_ specific avoided emissions (kg CO_2_/MW_el,net_ h)	-	916.61	788.21	916.61	836.77	916.61
Membrane area (m^2^)	-	-	413,000	409,000	562,000	530,000
**J_O2_ permeation rate (10^−6^ mol/cm^2^·s)**	-	-	1.32	1.33	1.02	1.19
Specific membrane area (m^2^/kW_el,net_)	-	-	0.62	0.64	0.85	0.69

**Table 8:** Economic results according to power plant configurations.

Economic Values	Reference Case	Case 1	Case 2	Case 3	Case 4	Case 5
Total Capital Investment (TCI)_M$_2020	1250	2018	1955	1708	1845	1944
Specific capital cost ($/kW_el,net_)	1523	3056	2942	2654	2779	2520
Annualized Total Capital Investment (TCIa)_M$_2020/y	118	190	185	161	174	183
Direct Annual Costs (DAC)_M$ 2020/y	134	140	141	140	140	146
Indirect Annual Costs (IAC)_M$ 2020/y	15	16	16	16	16	16
LCOE ($_2020/MWh)	44	71	70	67	68	61
C_cap_ ($2020/t)	-	23.10	21.98	18.73	19.79	16.57
C_av_ ($2020/t)	-	30.21	29.56	25.13	26.29	18.55

## Data Availability

The data are not publicly available due to restrictions of privacy.

## Abbreviations

MC	Multi-compressor system
ASU	Air Separation Unit
BL	Pressure mechanical devices
BAT	Best Available Techniques
CC	Combustor Chamber
CCS	Carbon Capture and Storage
CFB	Circulating Fluidized Bed boiler
COP-25	Conference of Parties held in Madrid
DAC	Direct Annual Cost
De-NO_x_	De-nitrification unit
De-SO_x_	De-sulphuration unit
DOE	United States Department of Energy
ECO	Economizer (water pre-heater)
FM	Fabric Filter
GHG	Control of Greenhouse Gas
HF	Hot Filter
HP	High-pressure Turbines
HX	Temperature exchange equipment
IAC	Indirect Annual Cost
IEA	International Energy Agency
IP	Intermediate pressure Turbines
IPPC	Intergovernmental Panel on Climate Change
LCOE	Levelized Cost of Electricity
LP	Low-pressure Turbines
OTM	Oxygen Transport Membrane
RH	Reheater
SH	Super-heater
SPCCC	Specific Energy Consumption for CO_2_ captured
SR	Oxygen separation ratio
TCI	Total Capital Investment
TPC	Total Production Cost
TG	Turbine Gas
VP	Vacuum Pump
Symbols	
C_Wagner_	Wagner conductivity constant, mol/cm·s·K
d_mem_	Membrane thickness, m
J_O2_	Oxygen permeation rate, mol/m^2^·s
F	Faraday’s constant, C/mol
K_Wagner_	Wagner temperature constant, K
y_O2_	oxygen molar fraction
m	Molar flow, mol/s
P	Total pressure, bar
$P_{O_{2}}$	Oxygen partial pressure, bar
R	Ideal gas constant, J/mol·K
T	Absolute temperature, K
Greek symbols	
σ	conductivity, S/m^2^
$\pi_{\mathrm{Mem}}$	Oxygen partial pressure ratio of membrane, dimensionless
Indices	
a	Air
boiler	boiler
el	Electronic
i	Ionic
f	Feed side
memb	Membrane
perm	Permeate side
ret	Retentate side
t	Theoretical
